# Recurrent erythema multiforme after alcohol ingestion in a patient receiving ciprofloxacin: a case report

**DOI:** 10.4076/1757-1626-2-7787

**Published:** 2009-07-16

**Authors:** Emmanuel Lagoudianakis, Apostolos Pappas, Nikolaos Koronakis, Ioannis Dallianoudis, Katerina Kotzadimitriou, John Chrysikos, Ilias Koukoutsis, Pantelis Antonakis, Dimitrios Keramidaris, Andreas Manouras

**Affiliations:** 1 2nd Department of Surgery, 417 NIMTS (Military Veterans' Fund Hospital)Monis Petraki 10, 11521, AthensGreece; 2 1st Department of Propaedeutic Surgery, Hippocrateion Hospital, Athens Medical SchoolQ. Sophia114, 11527, AthensGreece

## Abstract

The incidence of cutaneous adverse reactions to quinolones is low; moreover their development in patients with concomitant alcohol consumption is a phenomenon that has been scarcely reported. We present a case of 46-year-old male who developed erythema multiforme after ingestion of alcohol, while being treated with ciprofloxacin. The lesion was self-limiting and abstinence from alcohol permitted the completion of the course of therapy without any other adverse reaction.

## Introduction

Quinolones are relative safe and well tolerated drugs with therapeutic indications evolving infections of almost all body compartment [1]. The toxicity of these antimicrobial agents often involves the musculoskeletal and the central nervous system [2]. The dermatologic adverse reactions occur in approximately 1% of the patients and they include a broad range of effects, such as exanthema, photosensitivity and pruritus [3]. Erythema multiforme (EM) is an acute inflammatory skin reaction that has rarely been associated with quinolone treatment. To date, evidence regarding the development of this rare adverse reaction is mainly drawn from case reports and single-arm series with small patient numbers [4,5]. A review of the adverse cutaneous events related to ciprofloxacin treatment in Sweden between 1988 and 2000, revealed 116 cases, only two of which were diagnosed with EM [5]. Among the few reports that have been published, only one has documented the development of skin reaction to ciprofloxacin after alcohol consumption [6]. We describe a case of a patient who developed EM after ingestion of alcohol, while being treated with ciprofloxacin.

## Case presentation

A 46-year-old Caucasian Greek male presented to our department complaining about recurrent episodes of a bullous erythema in the pretibial region. Two weeks previously the patient was diagnosed with chronic pelvic pain syndrome and was commenced on a four-week trial with ciprofloxacin (500 mg PO every 12 hours). During the second week of treatment he noticed the presence of the skin reaction after the ingestion of alcohol (5%). The lesion was self-limiting lasting for few hours but recurred after alcohol consumption. The patient did not receive any other medication during that period and reported no previous history of drug allergy. Furthermore, although he described a long history of alcohol consumption he reported no adverse reaction. Subsequent physical examination and routine laboratory tests were normal. A skin biopsy was performed and showed epidermal necrosis with individual dyskeratotic keratinocytes, and a perivascular infiltrate of lymphocytes; histological features consistent with the diagnosis of EM. Abstinence from alcohol permitted the remission of the bullous erythema and the completion of the course of ciprofloxacin without any other adverse reaction.

## Discussion

Erythema multiforme is an acute, self-limiting, mucocutaneous reaction characterized by target or iris lesions of the skin [7]. It may be episodic or recurrent with great variability in the interval between episodes. It tends to arise in the third and fourth decades of life and is rarely seen under the age of 3 years or over the age of 50 years [8]. Previously, the condition was thought to be part of a clinical spectrum of disease that also included two more severe forms, Stevens-Johnson syndrome and toxic epidermal necrolysis. Nonetheless, there is increasing evidence that the later two are distinct from EM, due to their contrasting clinical presentations and potential causes [9].

The development of EM has been associated with both infectious and drug triggers. Herpes simplex virus (HSV) is the most commonly identified cause. Previous studies have reported that approximately 70% of cases were precipitated by a preceding HSV infection [10,11]. Mycoplasma pneumonia and fungal infection are also commonly reported etiologies, especially in children [12]. The medications most often associated with EM are barbiturates, hydantoins, nonsteroidal anti-inflammatory drugs, penicillins and sulfonamides [13].

In our case the EM developed after the consumption of alcohol in a patient being treated with ciprofloxacin. After taking under consideration the absence of any obvious etiologic factors, the recurrence of patient’s skin reaction after alcohol ingestion and the complete remission after alcohol abstinence, we suspected the presence of a possible causal association between ciprofloxacin, alcohol ingestion and EM. Previous reports have documented the appearance of cutaneous reaction to ciprofloxacin [4,5,14,15] and among them one case described the onset of symptoms after the ingestion of ethanol [6].

As noted above the time sequence of alcohol ingestion and the onset of the skin reaction, probable is more than incidental. We could speculate that the interactions between ciprofloxacin and ethanol oxidising enzymes in the hepatic level could produce metabolites capable of triggering a hypersensitivity reaction such as EM. Furthermore, chronic alcohol consumption can alter the function of both cellular and humoral immune mechanisms [16]. Deregulation of these mechanisms has also been implicated in the development of EM [7,9]. Hence, our patient’s past and present history of alcohol use could render him more susceptible to EM.

In summary, physicians should always be alert for adverse drug reactions; rapid recognition and prompt withdrawal of the causative agent are crucial for reducing any further toxicity. Patients that are being treated with ciprofloxacin should better avoid alcohol; nevertheless, due to the absence of a clear association between the two agents no firm suggestions can be made.

## Abbreviations

EM: Erythema multiforme
HSV: Herpes simplex virus

## References

[bib-001] Van Bambeke F, Michot JM, Van Eldere J, Tulkens PM (2005). Quinolones in 2005: an update. Clin Microbiol Infect.

[bib-002] Leone R, Venegoni M, Motola D, Moretti U, Piazzetta V, Cocci A, Resi D, Mozzo F, Velo G, Burzilleri L, Montanaro N, Conforti A (2003). Adverse drug reactions related to the use of fluoroquinolone antimicrobials: an analysis of spontaneous reports and fluoroquinolone consumption data from three italian regions. Drug Saf.

[bib-003] Stahlmann R, Lode H (1999). Toxicity of quinolones. Drugs.

[bib-004] Isik SR, Karakaya G, Erkin G, Kalyoncu AF (2007). Multidrug-induced erythema multiforme. J Investig Allergol Clin Immunol.

[bib-005] Hällgren J, Tengvall-Linder M, Persson M, Wahlgren CF (2003). Stevens-Johnson syndrome associated with ciprofloxacin: a review of adverse cutaneous events reported in Sweden as associated with this drug. J Am Acad Dermatol.

[bib-006] Vaidyanathan S, Singh G, Sett P, Watt JW, Soni BM, Oo T (1999). Cutaneous adverse reaction to ciprofloxacin precipitated by ingestion of alcohol in a tetraplegic patient. Spinal Cord.

[bib-007] Al-Johani KA, Fedele S, Porter SR (2007). Erythema multiforme and related disorders. Oral Surg Oral Med Oral Pathol Oral Radiol Endod.

[bib-008] Huff JC, Weston WL, MG (1983). Tonnesen, Erythema multiforme: a critical review of characteristics, diagnostic criteria, and causes. J Am Acad Dermatol.

[bib-009] Williams PM, Conklin RJ (2005). Erythema multiforme: a review and contrast from Stevens-Johnson syndrome/toxic epidermal necrolysis. Dent Clin North Am.

[bib-010] Kokuba H, Imafuku S, Huang S, Aurelian L, Burnett JW (1998). Erythema multiforme lesions are associated with expression of a herpes simplex virus (HSV) gene and qualitative alterations in the HSV-specific T-cell response. Br J Dermatol.

[bib-011] Sun Y, Chan RK, Tan SH, Ng PP (2003). Detection and genotyping of human herpes simplex viruses in cutaneous lesions of erythema multiforme by nested PCR. J Med Virol.

[bib-012] Ayangco L, Rogers RS (2003). Oral manifestations of erythema multiforme. Dermatol Clin.

[bib-013] Volcheck GW (2004). Clinical evaluation and management of drug hypersensitivity. Immunol Allergy Clin North Am.

[bib-014] Kundu AK (2000). Ciprofloxacin-induced severe cutaneous reaction and haemolysis in a young adult. J Assoc Physicians India.

[bib-015] Rönnau AC, Sachs B, von Schmiedeberg S, Hunzelmann N, Ruzicka T, Gleichmann E, Schuppe HC (1997). Cutaneous adverse reaction to ciprofloxacin: demonstration of specific lymphocyte proliferation and cross-reactivity to ofloxacin in vitro. Acta Derm Venereol.

[bib-016] Díaz LE, Montero A, González-Gross M, Vallejo AI, Romeo J, Marcos A (2002). Influence of alcohol consumption on immunological status: a review. Eur J Clin Nutr.

